# End to End Multitask Joint Learning Model for Osteoporosis Classification in CT Images

**DOI:** 10.1155/2023/3018320

**Published:** 2023-03-15

**Authors:** Kun Zhang, Pengcheng Lin, Jing Pan, Peixia Xu, Xuechen Qiu, Danny Crookes, Liang Hua, Lin Wang

**Affiliations:** ^1^School of Electrical Engineering, Nantong University, Nantong, Jiangsu 226001, China; ^2^Nantong Key Laboratory of Intelligent Control and Intelligent Computing, Nantong, Jiangsu 226001, China; ^3^Nantong Key Laboratory of Intelligent Medicine Innovation and Transformation, Nantong, Jiangsu 226001, China; ^4^Department of Radiology, Affiliated Hospital 2 of Nantong University, Nantong, Jiangsu 226001, China; ^5^College of Mechanical Engineering, Donghua University, Shanghai 201620, China; ^6^School of Electronics, Electrical Engineering and Computer Science, Queen's University Belfast, Belfast BT7 1NN, UK

## Abstract

Osteoporosis is a significant global health concern that can be difficult to detect early due to a lack of symptoms. At present, the examination of osteoporosis depends mainly on methods containing dual-energyX-ray, quantitative CT, etc., which are high costs in terms of equipment and human time. Therefore, a more efficient and economical method is urgently needed for diagnosing osteoporosis. With the development of deep learning, automatic diagnosis models for various diseases have been proposed. However, the establishment of these models generally requires images with only lesion areas, and annotating the lesion areas is time-consuming. To address this challenge, we propose a joint learning framework for osteoporosis diagnosis that combines localization, segmentation, and classification to enhance diagnostic accuracy. Our method includes a boundary heat map regression branch for thinning segmentation and a gated convolution module for adjusting context features in the classification module. We also integrate segmentation and classification features and propose a feature fusion module to adjust the weight of different levels of vertebrae. We trained our model on a self-built dataset and achieved an overall accuracy rate of 93.3% for the three label categories (normal, osteopenia, and osteoporosis) in the testing datasets. The area under the curve for the normal category is 0.973; for the osteopenia category, it is 0.965; and for the osteoporosis category, it is 0.985. Our method provides a promising alternative for the diagnosis of osteoporosis at present.

## 1. Introduction

Osteoporosis (OP) is a disease characterized by impaired bone microstructure and decreased bone mineral density (BMD). With the acceleration of population aging, OP has become an increasingly serious global health problem [1]. Fragile fracture is the most serious complication of OP [2]. OP causes more than 8.9 million brittle fractures each year worldwide [3]. In the US, fragile fractures are more than four times more common than stroke, acute myocardial infarction, and breast cancer [4]. In several developed countries, osteoporotic fractures account for longer hospitalization time than these diseases according to a meeting of the World Health Organization [5]. By 2025, the number of fragility fractures is expected to increase from 3.5 million in 2010 to 4.5 million, a 28% increase [6]. Therefore, reliable technology for the early detection and prevention of OP is urgently needed.

Currently, although dual-energyX-ray absorptiometry (DXA) is the gold standard for measuring bone mineral density for the diagnosis of OP, it is not widely used as a screening tool for OP owing to its high cost and limited availability of equipment [7]. To overcome these limitations, a variety of osteoporosis screening tools have emerged. Quantitative ultrasound (QUS) is one of them, which has developed into an alternative method for DXA screening of osteoporosis. Its benefits include being portable and economical; however, it may be unavailable in all primary medical settings [8]. In addition, a variety of clinical risk assessment tools have been developed to predict osteoporosis, including the fracture risk assessment tool (FRAX), the QFracture algorithm, the Garvan Fracture Risk Calculator, and the osteoporosis self-assessment tool [9]. Unfortunately, these tools are based on a combination of known risks to calculate the risk of fracture in patients and have poor efficiency.

Artificial intelligence and machine learning algorithms have recently been used in the diagnosis and prediction of osteoporosis [10]. The existing methods have achieved some success in solving the problem of binary classification (osteoporosis and nonosteoporosis) of which the main purpose is to identify whether the patient has osteoporosis [11]. However, these methods also have some obvious shortcomings: (1) the existing artificial intelligence algorithms treat segmentation and classification as two separate tasks, ignoring the information fusion and complementarity between the two tasks; (2) taking the average of two lumbar cancellous bone mineral density measurements (commonly the first and second lumbar) is widely acknowledged as the best diagnostic criterion for osteoporosis in lumbar QCT [12]. In current models, these data inputs tend to be CT images of a single vertebral body, disregarding the information fusion and complementarity between multiple vertebral images; (3) the problem of class imbalance in the collected data is prevalent due to the lack of standard public datasets; (4) most methods treat osteoporosis as a binary problem, regardless of the urgent need and a strong incentive to turn the binary into a trinomial (osteoporosis, osteopenia, and normal) problem. Although the three classifications are more difficult, osteopenia can bring some predictability to the prevention and treatment of osteoporosis. In this paper, we address the challenges above in the diagnosis of osteoporosis to facilitate the timely detection of the condition and propose an instance-based and class-based multilevel joint learning framework for bone state classification. The innovation of this method lies in the following steps. Firstly, we locate a vertebral body and remove redundant information from the image. Secondly, by constructing the boundary heat map regression auxiliary branch, the vertebral edge is refined, and the segmentation performance is improved on the segmentation branch of the shared encoder. In addition, low-level and high-level features from the segmentation branch and the auxiliary branch, including the shape and boundary of the vertebral body, are fused with feature layers from the diagnostic classifier. Finally, considering the different effects of different vertebral bodies on the classification results of bone state, we design a feature fusion module to adaptively learn feature fusion weights. The proposed method is novel because it solves the challenges of high dimensionality, multimodality, and multiclassification associated with osteoporosis diagnosis, and these challenges have not been resolved in earlier methods. The contributions of the research are as follows:

- A joint learning framework is proposed to segment vertebral bodies from CT images and classify bone states (normal, osteopenia, and osteoporosis)
- An instance-based and class-based data sampling balancing strategy is introduced to solve the problem of poor model prediction caused by imbalanced data between training datasets
- A boundary heat map regression branch is proposed, which uses the Gaussian function to do “soft labeling,” accelerating network convergence and improving the performance of vertebral segmentation in joint learning and single-task learning environments
- The effectiveness of segmentation features in guiding a deep classification network is verified by hierarchically fusing the features of the decoder and classifier related to two segmentation tasks
- A feature fusion module is proposed to adaptively learn the feature weights of vertebrae 1 and 2 and balance the influence of two vertebrae images on classification results

To our knowledge, there are many studies [13–16] on the classification of bone status using vertebral images, but there are few studies on multitask joint learning and detection of bone status based on soft tissue window images at the central level of lumbar 1 and lumbar 2 vertebrae. Experimental results show that multitask joint learning can improve the accuracy of disease classification.

## 2. Related Works

In this section, we briefly review the related research on bone state classification, categorizing them into three subareas to introduce the current research on the bone state in the medical image, i.e., vertebral positioning, vertebral CT image segmentation, and vertebral medical CT image classification.

### 2.1. Vertebral Positioning

With the development of deep learning, convolutional neural networks are increasingly used for positioning tasks. However, most of these works describe vertebral recognition as a centroid point detection task. Chen et al. used the advanced features of convolutional neural networks to represent vertebrae from 3D CT volume and eliminated the detection of misplaced centroids based on a random forest classifier [17]. Dong et al. iterated the centroid probability map of a convolutional neural network using a message-passing scheme according to the relationship between the centroids of the vertebrae and used sparse regularization to optimize the localization results to obtain a pixel-level probability of each vertebral centroid [18]. However, it may be more meaningful to directly identify the labels and bounding boxes of vertebrae (rather than the probability map of the centroid point). Zhao et al. proposed a category-consistentself-calibration recognition system to accurately predict the bounding boxes of all vertebrae, improving the discrimination ability of vertebrae categories and the self-awareness of false positive detection [19]. All of these methods identify the vertebrae from the coronal plane, whereas what we want is to get a small image from the transverse view that only contains the vertebrae.

### 2.2. Vertebral Segmentation

Recently, machine learning is increasingly used in the recognition and segmentation of vertebral bodies. Michael Kelm et al. used iterative variants of edge-space learning to find the bounding boxes of intervertebral discs and utilized Markov-based random fields and graphical cutting to initialize and guide the segmentation of the vertebrae [20]. Zukić et al. employed the AdaBoost-based Viola–Jones object detection framework to find the bounding boxes of the vertebrae and then split them by expanding the mesh from the center of each vertebra [21]. Chu et al. applied random forest regression to detect the vertebral center and used these to define target regions for the segmentation of the vertebrae with random forest voxel classification [22]. Although these methods can find certain vertebral bodies with specific appearances, they still need to set some parameters empirically and fail to deal with complex pathological cases. However, many recent segmentation methods are based on deep learning, using convolutional neural networks instead of the traditional explicit modeling of spine shape and appearance. For example, Sekuboyina et al. used a multiclass convolutional neural network for pixel labeling, segmented the lumbar spine on a 2D facet slice, and estimated the bounding boxes of the waist region using a simple multilayer perceptron to identify regions of interest in the graph [23]. Janssens et al. depended on two continuous networks to realize this task. First, they used a regression convolutional neural network to estimate the bounding box of the lumbar region and then used a classification convolutional neural network to perform voxel labeling in the bounding box to segment the vertebral body [24]. Mushtaq et al. used ResNet-UNet to semantically segment the lost vertebral body, achieving 0.97 DSC and 0.86 IOU [25].

### 2.3. Vertebral Medical Image Classification

In the study of establishing the osteoporosis model, Yoo et al. established a support vector machine model using age, height, weight, body mass index, hypertension, hyperlipidemia, and other factors to identify osteoporosis in postmenopausal women. Compared with traditional osteoporosis self-assessment tools, they found that the support vector machine model is more accurate [26]. Pedrassani de Lira et al. established a J48 decision number model to identify osteoporosis through multiple indicators such as age, previous fracture, number of previous fractures, and previous spinal fractures [27]. Tafraouti et al. extracted features from X-ray images and used a support vector machine model to identify osteoporosis, which can well distinguish osteoporosis patients from normal people [28]. Kilic and Hosgormez studied the identification of osteoporosis based on a random subspace method and random forest ensemble model. Jang et al. used a deep learning method to identify osteoporosis [29]. In the internal and external test sets, the area under curve (AUC) of osteoporosis screening was 0.91 (95% confidence interval (CI), 0.90–0.92) and 0.88 (95% confidence interval (CI), 0.85–0.90), respectively. The experimental results illustrate that the use of chest radiographs based on deep learning models may be used for opportunistic automatic screening of osteoporosis patients in the clinical environment [30]. In the latest study, Xue et al. conducted a study in which they labeled the L1–L4 vertebral body in CT images and divided it into three categories based on bone mineral density: osteoporosis, osteopenia, and normal. The study achieved a high level of accuracy, with a prediction accuracy of 83.4% and a recall rate of 90.0% [31]. Dzierżak and Omiotek have developed a novel method for diagnosing osteoporosis through the use of spine CT imaging and deep convolutional neural networks. To address the issue of a small sample size, they utilized a large dataset to pretrain their model, which resulted in the successful classification of osteoporosis and normal cases. This approach showed promising results for the accurate diagnosis of osteoporosis using CT scans [32]. In these methods, both the traditional machine learning algorithm and the current popular deep learning algorithm use the image containing only the region of interest as the data source. The step-by-step preprocessing process is tedious, time-consuming, and inefficient. Therefore, the integration of positioning, segmentation, and classification into a network should help to improve efficiency, and no research has shown that explicit or implicit features related to the first 3/4 of the vertebral body can be effectively and interpretably used in deep classification networks.

## 3. Proposed Methods

### 3.1. Overview

**Figure 1:**
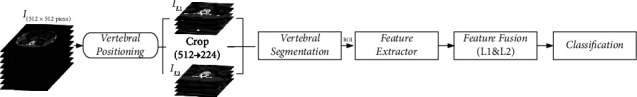
Joint framework scheme, including vertebral positioning module and vertebral segmentation module, combined with gated attention cascade feature extraction module and feature fusion module (L1 and L2).

**Figure 2:**
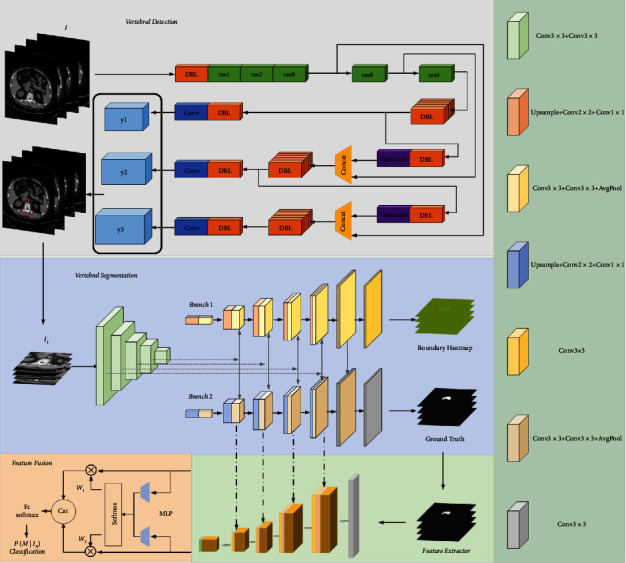
Joint framework scheme specific network architecture, including (i) the CT image is sent to YOLOv3 for vertebral positioning; (ii) then, the segmentation module is used to segment the region of interest of the vertebral body, and the feature maps of different scales of the decoding layer are cascaded with the features learned by the ResNet-based convolutional feature extractor, and the key features are obtained by modeling the context features through gated attention; (iii) finally, the features of L1 and L2 are fused by the feature fusion module, and the CT image is classified.

Our proposed method aims to classify vertebral images within a joint framework to enable a more flexible diagnosis of osteoporotic lesions. To achieve this goal, as shown in Figure 1, we propose an instance-based and class-based end-to-end multitask joint learning framework. It mainly has a strategy to solve class imbalance and four deep learning modules, including vertebral positioning module, vertebral segmentation module, cascade feature extraction module combined with gated attention, and feature fusion module. As shown in Figure 2, a new multilayer and multilevel joint learning framework is introduced, which integrates positioning, segmentation, and classification. Firstly, realizing the accurate location of the target lesion (coronal vertebral body), removing the redundant information of the image through the reduction of resolution (from 512 × 512 to 224 × 224). Secondly, the boundary heat map auxiliary branch is employed to refine the edge to improve the performance of segmentation; meanwhile, segmentation features are cascaded with the classification features to improve the accuracy of classification. Finally, we propose a feature fusion module, which adaptively assigns feature weights to fuse the features of lumbar L1 and lumbar L2. Different magnitudes of losses in multitask learning tend to bring about negative effects on other tasks when the model tends to fit a certain task; to balance this problem, we use the gradient update method to assign weights to each loss, exploiting neural networks to update the weight parameters.

### 3.2. Instance and Class-Based Sampling Methods

In the actual clinical scene, the data collected by image acquisition will be unbalanced owing to the inherent difficulty of collecting labels of rare diseases or other unusual cases. Therefore, when training on extremely unbalanced data, the model may have a high probability of being affected by the number of different categories, resulting in the underfitting of some categories which may be ignored. At present, the methods to solve the data imbalance include data resampling [33], adaptive loss function [34], and curriculum learning [35]. Inspired by the paper [36, 37], methods are introduced to solve the problem of extreme imbalance of our category images. It combines unbalanced (instance-based) and balanced (class-based) sampling of data, where we extend the method to our three-category practical problems.

We define the training set as *D*={(*x*_*i*_, *y*_*i*_), *i*=1,2,…, *N*}, where *x*_*i*_ is the sample, *y*_*i*_ is the sample category. Assuming that for multiclassification problems with *K* categories, each category has *M*_*k*_ samples, and *N* represents the total number of samples, where ∑_*K*=1_^*K*^*M*_*k*_=*N*, the general sampling strategies can be described as

(1) $$\begin{array}{c} p_{j}=\frac{M_{j}^{n}}{\overset{k}{\underset{k=1}{\sum }}M_{j}^{n}}\text{,} \end{array}$$

where *p*_*j*_ is the probability of sampling from the *j* th category. If we set *n*=0, the probability of sampling from each category is equal to 1/*K*. This is the class-based sampling method.

**Figure 3:**
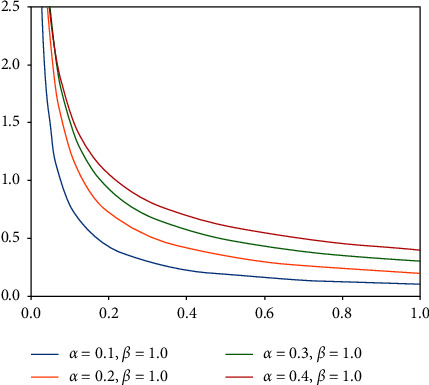
Beta(*α*, 1) distribution for varying *α*.

If we set *n*=1, then it is equivalent to selecting the sample by the proportion of a category of samples to all samples, which is instance-based sampling. Here, we introduce a mixed sampling method based on instance and class, which is suitable for data imbalance. We denote the training dataset and sampling strategy by the symbol (*D*, *S*). Instance-based sampling and class-based sampling are represented by *S*_*I*_ and *S*_*C*_, respectively, so this mixture can be described as

(2) $$\begin{array}{c} \overset{\wedge }{x}=\lambda x_{I}+\left(1-\lambda \right)x_{C}\text{,}\\ \overset{\wedge }{y}=\lambda y_{I}+\left(1-\lambda \right)y_{C}\text{,} \end{array}$$

where *λ* ~ beta(*α*, *β*), *α* > 0, *β* > 0, *λ* ∈ [0,1], (*x*_*I*_, *y*_*I*_) ∈ (*D*, *S*_*I*_), (*x*_*C*_, *y*_*C*_) ∈ (*D*, *S*_*C*_). $\overset{\wedge }{x}$ and $\overset{\wedge }{y}$ represent random convex combinations of data and label inputs. Here, we set *β*=1. As shown in Figure 3, as *α* grows, examples from minority classes are combined with a greater weight to avoid overfitting of minority classes. Here, we set *α*=0.1 to induce a more balanced distribution of training samples by creating synthetic data points around spatial regions where minority classes provide fewer data density.

### 3.3. Vertebral Positioning Module Based on YOLOv3

The basic step of vertebral CT image classification is to extract robust features from CT images, given *W* and *H* of the original images are 512 pixels. To remove redundant features, we use the YOLOv3 [38] to locate the vertebral body in the image with size 512 × 512 × 3 as input to YOLOv3. The image feature is extracted by DarkNet-53, and then the target classification and position regression are performed on the acquired feature map with the help of the FPNs (feature pyramid networks) structure.

In this study, we will obtain the position of the prediction box in the original image *p*_*x*_, *p*_*y*_, *p*_*w*_, *p*_*h*_, in YOLOv3, a set of anchor frames is composed of nine initial frames of different sizes. Assuming that the center coordinates, width, and height of an anchor frame are expressed as *a*_*x*_, *a*_*y*_, *a*_*w*_, *a*_*h*_, *p*_*x*_, *p*_*y*_, *p*_*w*_, *p*_*h*_ can be obtained by reverse calculation of the regression parameter *t*_*x*_, *t*_*y*_, *t*_*w*_, *t*_*h*_ by the output network. Details of the calculation formula are as follows:

(3) $$\begin{array}{c} p_{x}=\sigma \left(t_{x}\right)+a_{x}\text{,}\\ p_{y}=\sigma \left(t_{y}\right)+a_{y}\text{,}\\ p_{w}=a_{w}e^{t_{w}}\text{,}\\ p_{h}=a_{h}e^{t_{h}}\text{,} \end{array}$$

where *σ*(*·*) represents the sigmoid transformation of the variable, aiming at controlling the offset of the center point between 0 and 1.

The main purpose of employing YOLOv3 is to obtain the center coordinates *p*_*x*_ and *p*_*y*_ of the prediction box and utilize this position as the center cutting position of the vertebral body to obtain a 224 × 224 image containing the complete vertebral body as the input of the subsequent convolution module. In this way, we can remove tens of thousands of useless features and improve the efficiency of the model.

### 3.4. Boundary Regression Auxiliary Branches

We suggest dividing the segmentation task into two tasks: vertebral segmentation and contour determination. Thus, our network is mainly composed of a weight-sharing encoder and two decoders composed of the segmentation branch and boundary regression branch. In the encoder, we improve the original U-Net [39] by applying residual blocks to replace the original two effective convolutions. In the decoder stage, we cascade the penultimate features from the boundary regression branch with the penultimate features of the segmentation branch, helping the network to better perceive and refine the vertebral contour. Since vertebrae in CT images may show up hyperosteogeny or other conditions, it is necessary to reconstruct edges by constructing auxiliary tasks, which provide more explicit and implicit topological priors for the coding layer and enable them to assist with the segmentation branches to obtain more accurate target masks.

The problem of boundary inaccuracy is rooted in the similarity of information in the corresponding receptive field of pixels. When similar features belong to the interior or exterior of the segmented region, this similarity will be advantageous, inversely similar information lies in the segmented boundary will undoubtedly increase the uncertainty of the edge. In terms of the boundary regression auxiliary branch in the segmentation module, we propose to divide the edge based on the region and graph from the whole image, combining it with the spatial proximity and pixel value similarity. In this paper, the accurate boundary of vertebral segmentation should be the inner boundary. We combine the convolutional neural network with the level set, taking the segmentation result obtained by the neural network as the prior knowledge of level set segmentation; then we construct a gray level constraint term on the original level set function and improve the edge indicator function to deal with uneven intensity in the image.

#### 3.4.1. Improve the Edge Indicator Function

Getreuer [40] proposed the famous Chan–Vese (CV) model in 2001. This method uses a region-based segmentation strategy to divide the image into two homogeneous regions, the inner and outer regions, using active contoured lines to find the image to be segmented and the original image with the minimum difference to minimize the energy function.

Given the input image *I*(*x*, *y*), the energy function based on the CV is shown as follows:

(4) $$\begin{array}{c} E\left(C,C_{1},C_{2}\right)=\mu \int_{\Omega }g\delta \left(\phi \right)\left|\nabla \phi \right|dx\;dy\\ +\upsilon \int_{\Omega }gH\left(-\phi \right)dx\;dy\\ +\lambda_{1}\int_{\Omega_{1}}{\left|I-C_{1}\right|}^{2}dx\;dy\\ +\lambda_{2}\int_{\Omega_{2}}{\left|I-C_{2}\right|}^{2}dx\;dy\text{,} \end{array}$$

where *C*_1_ and *C*_2_ describe the average gray levels of equivalent parts inside and outside the contour, respectively, Ω_1_ and Ω_2_ represent the inner and outer regions of the contour, *λ*_1_, *λ*_2_, *μ*, *υ* are constants, *g*=(1/1+|∇*G*(*x*, *y*, *σ*)*∗I*(*x*, *y*)|) is the edge indicator function which can be used to prevent the curve from exceeding the target area, *G* is the Gaussian calculation sub, *σ* is the standard deviation, and *δ* and *H* represent Dirac and Heaviside functions, respectively. The position of contour *C* and unknowns *C*_1_(*ϕ*) and *C*_2_(*ϕ*) are finally obtained through optimization formula (4).

The evolution of the CV model is constrained by global gray-level information. However, most images, especially medical images, have uneven intensity. To solve this problem, we improve the function *g* and construct gray-level information constraint terms to constrain the evolution direction. Bilateral filtering is a method that combines the spatial proximity of images with the similarity of pixel values. Based on Gaussian filtering, bilateral filtering introduces the gray value of pixels for the local weighted average. When smoothing the speckle noise of images, bilateral filtering can better maintain the edge features.

In the first step, the Gaussian function *G*_*sr*_(*x*, *y*, *σ*) is used to construct bilateral filters to obtain smooth images:

(5) $$\begin{array}{c} G_{sr}\left(x,y,\sigma \right)=G_{\sigma s}*G_{\sigma r}\text{,}\\ G_{\sigma s}=e^{\left(-{\left(x-k\right)}^{2}+{\left(y-l\right)}^{2}/2\sigma_{s}^{2}\right)}\text{,}\\ G_{\sigma r}=e^{\left(-{\left\|I\left(x,y\right)-f\left(k,l\right)\right\|}^{2}/2\sigma_{r}^{2}\right)}\text{.} \end{array}$$

Image *I*(*x*, *y*) is filtered using bilateral filter operator *g*(*x*, *y*)=*G*_*sr*_(*x*, *y*, *σ*)*·I*(*x*, *y*), where *σ*_*r*_ is the standard deviation used to control the smoothness, *i*, *j*, *k*, *l* are the weight coefficients.

In the second step, the optimal threshold *T* is calculated based on the filtered image using the adaptive threshold principle. The maximized interclass variance value of *T* is shown in the following equation:

(6) $$\begin{array}{c} \nu^{2}=w_{0}\times w_{1}\times \left(u_{0}-u_{1}\right)\text{,} \end{array}$$

where *w*_0_ represents the ratio of pixels in the target area to the image, *u*_0_ represents the corresponding average gray level, *w*_1_ is the proportion of background pixels, and *u*_1_ is the average gray level of background pixels. Then, the new edge indicator function *g*_*r*_ can be described as

(7) $$\begin{array}{c} g_{T}=\frac{1}{1+\nu^{2}\left|\nabla G_{sr}\left(x,y,\sigma \right)*I\left(x,y\right)\right|}\text{.} \end{array}$$

#### 3.4.2. Auxiliary Branch

We advocate the segmentation results of convolutional neural networks as prior knowledge, namely, the initial contour of the level set, and the curve contour evolved through the level set is used to guide the neural network to optimize toward the edge of the vertebral body.

The specific expression of the gray level constraint *Q* is described as

(8) $$\begin{array}{c} Q=\gamma \left[\frac{1+\Gamma }{2}-\frac{1-\Gamma }{2}\right]H\left(\phi \right)\text{,}\\ \Gamma =\left\{\begin{array}{cc} -1\text{,} & I\in \left(I_{low},I_{high}\right)\text{,}\\ 1\text{,} & I\notin \left(I_{low},I_{high}\right)\text{,} \end{array}\right.\\ I_{low}=\eta -w\cdot \sigma \text{,}\\ I_{high}=\eta +w\cdot \sigma \text{,} \end{array}$$

where *I*_high_ is the upper limit of the vertebral gray value obtained by using the convolutional neural network model, *I*_low_ is the lower limit of vertebral gray value, *σ* is the average of vertebral gray value, *η* is the variance of vertebral gray value, and *w* is a constant.

The function of the gray level information constraint term is to make the level set curve evolve inside the vertebral body to approximate the inner edge contour. When the gray value of the pixel is within the upper and lower limits of the initial vertebral gray value, the energy value of the point is negative, otherwise positive. The edge result obtained by the neural network is used to replace *x* and *y* on the initial contour plane. Gradient descent is used to minimize the energy function, and the formula form of the final evolution equation after adding the gray constraint function is shown as follows:

(9) $$\begin{array}{c} \frac{\partial \phi }{\partial t}=\delta \left(\phi \right)\left[\begin{array}{c} \mu \mathrm{div}\left(g_{T}\frac{\nabla \phi }{\left|\nabla \phi \right|}\right)-g_{T}\upsilon \\ -\lambda_{1}{\left[I\left(x,y\right)-C_{1}\right]}^{2}+\gamma \\ \left[\frac{1+\Gamma }{2}-\frac{1-\Gamma }{2}\right]+\lambda_{2}{\left[I\left(x,y\right)-C_{2}\right]}^{2} \end{array}\right]\text{,}\\ C_{1}\left(\phi \right)=\frac{\int_{\Omega }I\left(x,y\right)H\left(\phi \right)dx\;dy}{\int_{\Omega }H\left(\phi \right)dx\;dy}\text{,}\\ C_{2}\left(\phi \right)=\frac{\int_{\Omega }I\left(x,y\right)\left[1-H\left(\phi \right)\right]dx\;dy}{\int_{\Omega }\left[1-H\left(\phi \right)\right]dx\;dy}\text{,}\\ \phi_{0}=\phi \left(0,I\left(x,y\right)\right)\text{.} \end{array}$$

In the label aspect of the auxiliary branch, we use the Canny operator to detect the edge of the binary image label. Canny is built on a two-dimensional convolution. To improve the calculation speed of the Canny operator, two-dimensional convolution can be decomposed into one-dimensional filters, and then a convolution operation with the image *A*(*x*, *y*) is carried out, respectively: *E*_*x*_=(*∂G*/*∂x*)*·A*(*x*, *y*), *E*_*y*_=(*∂G*/*∂y*)*·A*(*x*, *y*). Then, the gradient amplitude *A*(*x*, *y*) and gradient *a*(*x*, *y*) direction can be expressed as

(10) $$\begin{array}{c} A\left(x,y\right)=\sqrt{{E_{x}}^{2}\left(x,y\right)+{E_{y}}^{2}\left(x,y\right)}\text{,}\\ a\left(x,y\right)=\arctan\frac{E_{y}\left(x,y\right)}{E_{x}\left(x,y\right)}\text{.} \end{array}$$

The size of the Gaussian window is adjusted by changing the standard deviation *σ* of the Gaussian function, that is $A\left(x,y\right)=\max\left(\sqrt{E_{x}^{2}+E_{y}^{2}}\right)$. We first apply nonmaximum suppression, and then segment images through the dual-threshold method. When the gradient of some pixel is greater than the limit threshold, it will be considered as an edge pixel.

Then, we construct a soft label heat map in the form of Heatsum based on the processed images:

(11) $$\begin{array}{c} Heatsum\left(G\left(x_{1},y_{1},\sigma \right),G\left(x_{2},y_{2},\sigma \right)\right)=1-\left(1-G\left(x_{1},y_{1},\sigma \right)\right)\left(1-G\left(x_{2},y_{2},\sigma \right)\right)\text{,}\\ G_{bd}=Gaussheat\left(\partial G\right),\\ =Heatsum\left(G\left(x_{1},y_{1},\sigma \right),\ldots G\left(x_{n},y_{n},\sigma \right)\right),\forall_{G\left(x_{n},y_{n},\sigma \right)}\in \partial G\text{,} \end{array}$$

where ○ represents the Hadamard product; it is noted that *G*_*bd*_ is normalized between [0, 1].

Here, the boundary regression branch is utilized to refine the segmented edges. We treat this branch as a regression task through mean square error rather than a whole work consisting of a boundary segmentation task together with the segmentation branch.

### 3.5. Cascading Classification Module

**Figure 4:**
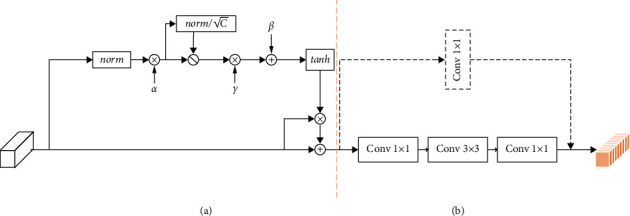
Gated residual module. (a) Gct layer. (b) Residual layer.

In the classification module, we use ResNet-101 as a basic feature extractor. ResNet [41] is a traditional deep convolutional neural network where the residual structure is used in the shallow network. The corresponding structure is illustrated in Figure 4(b). By adding the input value *x* with the output unit, the residual gains better performance in convergence after the operation of ReLU active. These steps can be approximated as an identical mapping of equal input and output, which effectively solves the problems of network learning ability decline, gradient disappearance, and gradient explosion when the number of convolutional neural network layers increases.

**Figure 5:**
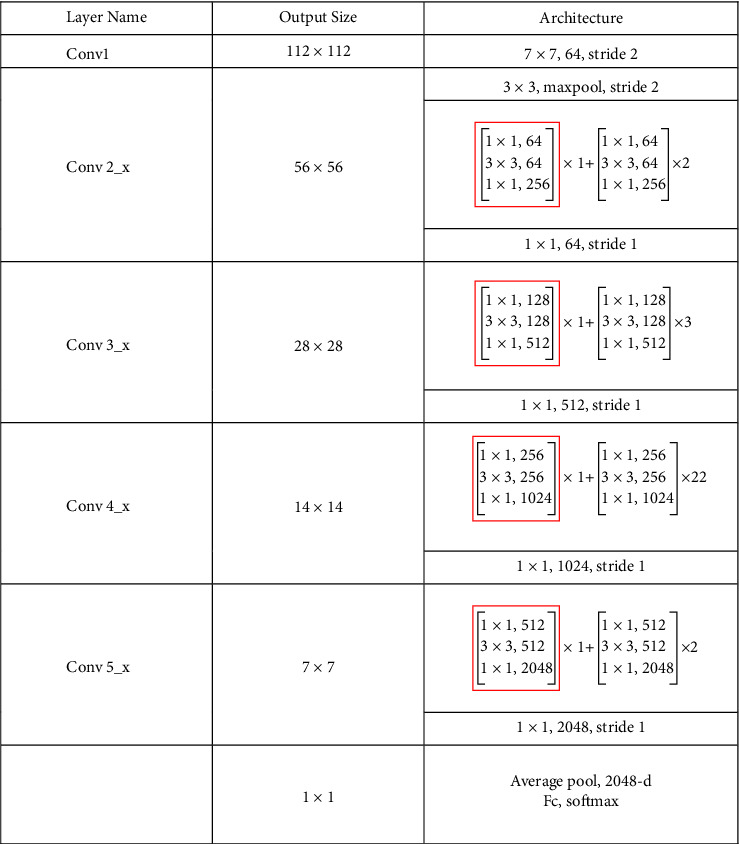
Feature extraction module network structure diagram. The convolution block framed in red is replaced by a gating residual module.

Inspired by the gating attention [42] and residual structure, we designed a gating residual module as shown in Figure 4 to replace the first convolution module in ResNet-101 from conv2_x to conv5_x. The specific network parameters can be found in Figure 5. The gated residual model can be described as follows.

Assuming that *x* ∈ *ℝ*^*C*×*H*×*W*^ is the activation feature of the convolutional neural network, where *H* and *W* are the height and width of the image, and *C* is the number of channels of the image, in general, the gating attention performs the following transformation.

(12) $$\begin{array}{c} \overset{\wedge }{x}=F\left(x\left|\alpha ,\gamma ,\beta \right.\right),\alpha ,\gamma ,\beta \in R^{C}\text{.} \end{array}$$

Among them, *a*, *β*, and *γ* are trainable parameters. The embedding weight *a* is mainly responsible for adjusting the embedding output, and the gating weight *γ* and the bias weight *β* are responsible for adjusting the gating activation.

They determine the behavior of gated attention in each channel.

For the specific process, assuming the given embedding weight as *α*=[*α*_1_, *α*_2_ …, *α*_*c*_], modules can be defined as

(13) $$\begin{array}{c} s_{c}=\alpha_{c}{\left\|x_{c}\right\|}_{2}\\ =\alpha_{c}{\left\{\left[\overset{H}{\underset{i=1}{\sum }}\overset{W}{\underset{j=1}{\sum }}{\left(x_{c}^{i,j}\right)}^{2}\right]+\in \right\}}^{1/2}\text{,} \end{array}$$

(14) $$\begin{array}{c} \overset{\wedge }{s_{c}}=\frac{\sqrt{C}s_{c}}{{\left\|S\right\|}_{2}}\\ =\frac{\sqrt{C}s_{c}}{{\left[\left(\sum_{c=1}^{C}{s_{c}}^{2}\right)+n\right]}^{1/2}}\text{,} \end{array}$$

where ∈ is a small constant, which is mainly used to avoid the derivation of zeros. Equation (14) is used to normalize channels, and *n* represents a small constant. $\sqrt{C}$ is used for normalization the ratio of *s*_*c*_, preventing the condition of small *s*_*c*_ when *C* is too large, *α*_*c*_ is a trainable parameter used for controlling the weight of each channel. When *α*_*c*_ is close to 0, the channel will not participate in channel normalization.

Then, we suppose the selection weight *γ*=[*γ*_1_, *γ*_2_ …, *γ*_*c*_] and the gating offset *β*=[*β*_1_, *β*_2_ …, *β*_*c*_], the gating function can be depicted as follows:

(15) $$\begin{array}{c} \overset{\wedge }{x_{c}}=x_{c}\left[1+\tanh\;\left(\gamma_{c}\overset{\wedge }{s_{c}}+\beta_{c}\right)\right]\text{.} \end{array}$$

Each primitive channel *x*_*c*_ is adapted by the corresponding gate, *γ* and *β* are trainable weights and deviations which is used to control the activation of the gate. Finally,$\overset{\wedge }{x_{c}}=\left[\overset{\wedge }{x_{1}},\overset{\wedge }{x_{2}}\ldots ,\overset{\wedge }{x_{c}}\right]$ will be entered into the residual module to obtain the feature map *y*=[*y*_1_, *y*_2_ …, *y*_*c*_] of the gating attention after the convolution operation. Supposing the feature map concatenated from the segmentation module *S*=[*S*_1_, *S*_2_ …, *S*_*c*_], we can perform the following operations on the classification network feature *y*=[*y*_1_, *y*_2_ …, *y*_*c*_] and the segmentation module feature to obtain the final feature map $\overset{\wedge }{y_{c}}$.

(16) $$\begin{array}{c} \overset{\wedge }{y_{c}}={Conv}_{1\times 1}{\left(Concat\left(y_{c},S_{c}\right)\right)}_{i=1,2\ldots c}\text{.} \end{array}$$

Two 1 × 2048-dimensional feature vectors of vertebrae can be obtained by flattening the feature map.

### 3.6. Feature Fusion Module

As mentioned above, the detection of bone status is based on the average of lumbar L1 and lumbar L2. To explain the different effects of different lumbar vertebrae on classification, we learn *W*_1_ and *W*_2_ adaptively for each vertebra, which satisfies *W*_1_+*W*_2_=1; *W*_1_ and *W*_2_ represent the fusion weights, respectively.

(17) $$\begin{array}{c} X_{fuse}=Concat\left(W_{1}\times X_{1},W_{2}\times X_{2}\right)\text{.} \end{array}$$

Specifically, we calculate *W*_1_ and *W*_2_ (*W*_1_+*W*_2_=1) by *F*_fuse_(*X*_1_) and *F*_fuse_(*X*_2_), respectively, where *F* represents the perception of two layers, that is, two fully connected layers. The following *softmax* layer can be used to eliminate the influence of different feature dimensions. After gaining the feature *X*_fuse_, the prediction of bone state *P*(*M*|*I*_*N*_) can be given by the fully connected layer and softmax function.

(18) $$\begin{array}{c} P\left(\left.M\right|I_{N}\right)=softmax\left(fc\left(X_{\text{fuse}},num-classes\right)\right)\text{.} \end{array}$$

### 3.7. Cascading Classification Models

To balance the impact of different dimensions of multiple tasks in the training process we introduce the trade-off parameters *λ*_1_, *λ*_2_, *λ*_3_, *λ*_4_ and *λ*_5_ to balance these four tasks. The total loss function of multitask learning can be defined as

(19) $$\begin{array}{c} L_{mul}=\lambda_{1}L_{IOC}+\lambda_{2}L_{cla}+\lambda_{3}L_{conf}+\lambda_{4}L_{seg}+\lambda_{5}L_{seg}+\lambda_{6}L_{cla}\\ =\lambda_{1}L_{IOC}\left({p_{i}}^{I},t\right)+\lambda_{2}L_{sobj}\left(p_{i}^{c1},q_{cla1}\right)+\lambda_{3}L_{obj}\left(p_{i}^{c2},q\right)+\lambda_{4}L_{dice}\left(S\left({p_{i}}^{s}\right),G\right)+\\ \lambda_{5}L_{mse}\left({p_{i}}^{b},G_{bd}^{n}\right)+\lambda_{6}L_{crossentropy}\left(p_{i}^{c3},q_{cla2}\right)\text{,} \end{array}$$

where *p*_*i*_^*I*^, *p*_*i*_^*c*2^, *p*_*i*_^*s*^, *p*_*i*_^*s*^, *p*_*i*_^*b*^, *p*_*i*_^*c*3^, respectively, represent the predicted results of the positioning branch, category branch, confidence branch, and segmentation branch of the positioning module for a given input image, the boundary heatmap regression branch, and the classification network. *S* represents the Sigmoid function, *t* represents the prediction box result, and *q*_cla1_ is the result of the category in the positioning module. *q* represents the probability that a vertebral body exists, *G*_*bd*_^*n*^ represents the normalized result of *G*_*bd*_, and *q*_cla2_ is the expected result of the classification network.

## 4. Experimental Results

### 4.1. Dataset and Preprocessing

To assess the effectiveness and benefit of the joint learning framework in bone state classification, we conducted experiments in a dataset obtained from the Nantong First People's Hospital from May 2021 to May 2022, consisting of CT images of 1048 routine-dose cases. All images were collected by Ingenuity Core 128 CT (Philips Health Care, Holland), the tube voltage was 120 kV, the inpatient tube current modulation technique was used, and the iDose 4 was used to reconstruct the cross-sectional image of the mediastinal window (standard B standard reconstruction algorithm). The reconstruction layer thickness and layer interval were both 2 mm. The longitudinal window images of the lumbar 1 and lumbar 2 center planes of each subject were selected for BMD measurement and deep learning model construction. The QCT pro4 software (Mindways, CA, USA) was used to set the same size of the region of interest (ROI) in the central cancellous bone area of the lumbar 1 and lumbar 2 vertebral bodies, avoiding the cortical bone and the visible vascular area. The software automatically calculated the BMD values of the lumbar 1 and lumbar 2 vertebral bodies and used their mean values as the BMD values of the individual subjects (BMD individuals). According to the standard recommended by the “expert consensus on imaging and bone mineral density diagnosis of osteoporosis” BMD individuals > 120 mg/cm^3^ are normal bone mass, 80 mg/cm^3^ ≤ BMD individuals ≤ 120 mg/cm^3^ are osteopenia, and BMD individuals < 80 mg/cm^3^ are osteoporosis.

**Figure 6:**
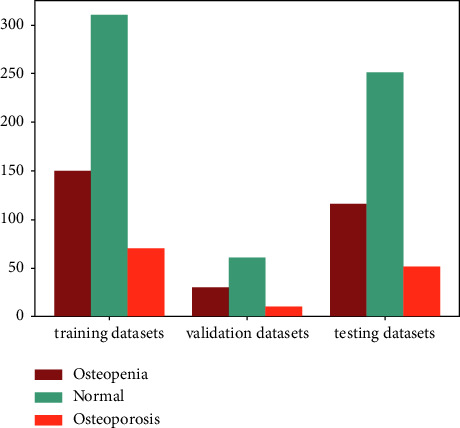
Data distribution of datasets.

We divide the dataset into training data (50%), validation data (10%), and test data (40%); the class distribution of training, validation, and testing datasets is shown in Figure 6. These three datasets do not have any overlapping images, and the CT images of each category in the three datasets are placed in strict proportions. Then, all images are resized to 512 × 512 and each image is normalized from [0, 255] to [0, 1] before being fed into the network.

To increase the amount of training data and improve the generalization ability and robustness of the model, we enhance the image data employing flipping, rotating, and scaling on the basis of the original data balancing strategy based on an instance and actual class.

### 4.2. Implementation of Framework

To implement the joint learning framework, we implemented the model based on Python 3.6.12, using the PyTorch framework and two NVIDIA GeForce 3090Ti GPUs. We apply the SGD optimizer to train the joint learning framework for 300 epochs with a learning rate of (10*e* − 1–10*e* − 5) and add six adaptive parameters to the SGD optimizer to weigh the loss of multitask learning.

### 4.3. Measurements and Baselines

#### 4.3.1. Measurements

Based on previous work[49–52], accuracy, sensitivity, specificity, and *F*1-score were used to evaluate the performance of classification. The accuracy rate is the ratio of the number of samples correctly classified by the classifier to the total number of samples. The sensitivity reflects the proportion of positive cases correctly judged by the classifier to the total positive samples. The specificity indicates the proportion of negative cases correctly judged by the classifier to the total negative samples. *F*1-score is the sum of accuracy and sensitivity. In this paper, the three-category problem is transformed into a two-category problem to evaluate; that is, the category studied at this time is a positive sample and the other categories are negative samples.

- Accuracy: Acc = (TP + FN/TP + TN + FP + FN)
- Sensitivity: Pre = (TP/TP + FN)
- Specificity: Spe = (TN/FP + TN)
- *F*1-score: F1 = (2 × P × R/P + R), *P* = (TP/TP + FP), *R* = (TP/TP + FN)

*P* denotes the model prediction and *T* denotes the true label. Positive samples are predicted as positive samples (true positive, TP), positive samples are predicted as negative samples (false negative, FN), negative samples are predicted as positive samples (false positive, FP), and negative samples are predicted as negative samples (true negative, TN).

Based on previous works [53–55], we use the intersection over union (IOU) and dice coefficient (Dice) to evaluate the effectiveness of our model segmentation task and use the average precision (AP) to evaluate the effectiveness of the positioning task.

- Intersection over union: IOU=(TP/TP+FP+FN)
- Dice coefficient: Dice=(2TP/2TP+FP+FN)

#### 4.3.2. Baselines

To demonstrate the performance of our federated framework model, we compared our work with popular machine learning and deep learning methods, including AlexNet [43], VGG-19 [44], GoogLeNet [45], ResNet [41], DenseNet [46], ShuffleNet [47], and EfficientNet [48].

### 4.4. Results

We use ten-foldcross-validation to calculate the average results and show the performance of the joint framework in Table 1. We set the learning rate of 10*e* − 1–10*e* − 5 to evaluate the classification performance of the joint framework in different situations. We used normal (osteopenia and osteoporosis) as a positive sample and other categories as negative samples, achieving an accuracy of 0.971, a sensitivity of 0.964, a specificity of 0.976, and an *F*1-score of 0.964. We achieved 0.933 in accuracy, 0.970 in sensitivity, 0.836 in specificity, and 0.954 *F*1-score when osteopenia was used as a positive sample and other categories (normal and osteoporosis) as a negative sample. When we used osteoporosis as a positive sample and other categories (normal and osteopenia) as negative samples, we achieved an accuracy of 0.957, a sensitivity of 0.962, a specificity of 0.922, and an *F*1-score of 0.975. The best performance is obtained by the learning rate of 10*e* − 3, indicating that the classification problem of bone state CT images can be effectively solved by adjusting the hyperparameters.

**Figure 7:**
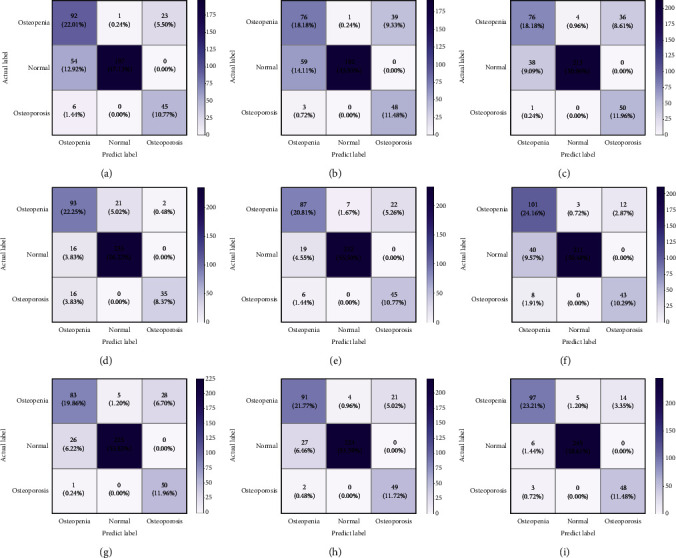
(a)–(h) The confusion matrix for each baseline method. (i) The confusion matrix for this method. (a) AlexNet. (b) VGG-19. (c) GoogLeNet. (d) ResNet-50. (e) ResNet-101. (f) DenseNet-121. (g) ShuffleNet. (h) EfficientNet. (i) Joint framework.

In addition, we compare the best results of joint learning with the most advanced baselines. The comparison results are reported in Table 2, where the best comparable performance is represented in bold. For the input images of other classification methods, we use CT images (512 × 512) generated by labels manually drawn by physicians that contain only regions of interest. To better intuitively compare the classification performance of the model, we use the confusion matrix for visual analysis. As shown in Figure 7, joint learning in dealing with the task of identifying low-dose achieves good performance with only 5 cases misclassified as normal, 2 cases misclassified as osteoporosis, and 8 cases misclassified as low doses; in the task of identifying osteoporosis, only 3 cases were misclassified as low dose. This result fully indicates the nonexistence of overfitting and underfitting states; this result further illustrates that there is no bias to a certain category which increases accuracy results.

**Figure 8:**
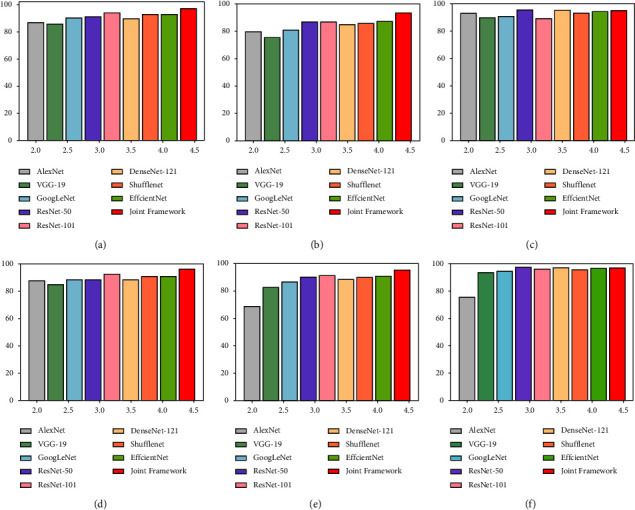
The classification performance comparison of each baseline method. (a) Normal-accuracy. (b) Osteopenia-accuracy. (c) Osteoporosis-accuracy. (d) Normal-*F*1-score. (e) Osteopenia-*F*1-score. (f) Osteoporosis-*F*1-score.

The histogram of accuracy and *F*1-score can be found in Figure 8. Intuitively, the accuracy rate has increased. Compared with the highest accuracy rate among advanced baseline methods, the accuracy rate of joint learning has increased by 6.2% in the osteopenia category, 3.3% in the normal category, and 0.1% in the osteoporosis category. Notably, when compared to the overall accuracy of advanced baseline methods, the overall accuracy of joint learning was improved by 3.8% which proved the effectiveness of joint learning strategies once again.

### 4.5. Further Discussion

#### 4.5.1. Roc Curve

**Figure 9:**
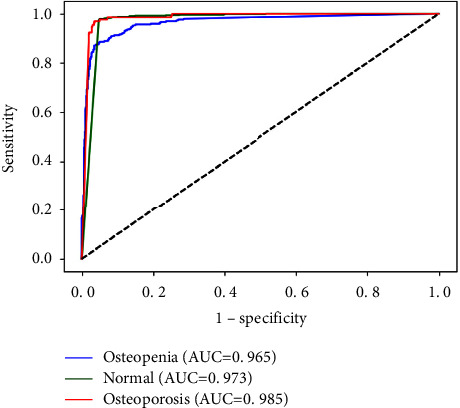
The ROC curve for learning rate 10*e* − 3.

To better demonstrate the classification ability of our proposed joint learning framework, we use the operating characteristic curve (ROC) and the area under curve (AUC) of receivers as further evaluation indicators. Taking the experimental results with a learning rate of 0.01 as an example, we draw the ROC curves of three categories in Figure 9, AUC for each category is also depicted in the figure. It can be found that the AUC in the osteopenia state is 0.965, the AUC value in the Normal state is 0.973, and the AUC value in the osteoporosis state is 0.985. These values prove the effectiveness of joint learning in bone CT image classification tasks.

#### 4.5.2. Training Convergence

**Figure 10:**
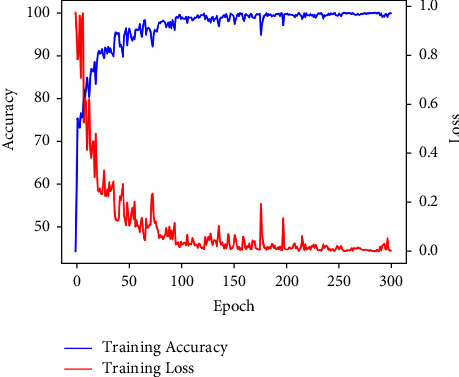
Accuracy and loss curve for learning rate 10*e* − 3.

For model training, we use the accuracy and loss curve and the training process to imply the training trend of accuracy and model cost. The accuracy and loss curves of the joint learning framework with a learning rate of 0.01 are shown in Figure 10, which reflects that the model's performance achieved satisfactory results at the 150th epoch and became stable. These two curves show the convergence of the model and assess its stability in bone CT image classification. In addition, the total training time of the joint learning framework on our dataset is about 10 hours, and each epoch takes 2 minutes. In short, training convergence and time reveal the computational efficiency of our network.

#### 4.5.3. Model Visualization

**Figure 11:**
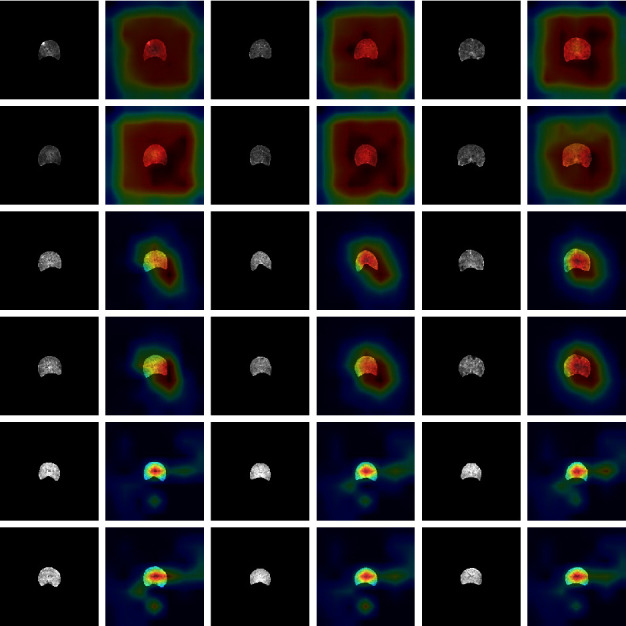
Grad-CAM visualization of 9 cases. It can be seen that different categories of networks have different emphases, which can be used as an explanation of neural networks. The first line of each two lines represents the L1 vertebrae, and the second line represents the corresponding L2 vertebrae. The first two lines represent osteoporosis cases, the middle two lines represent osteopenia cases, and the last two lines represent normal cases.

**Figure 12:**
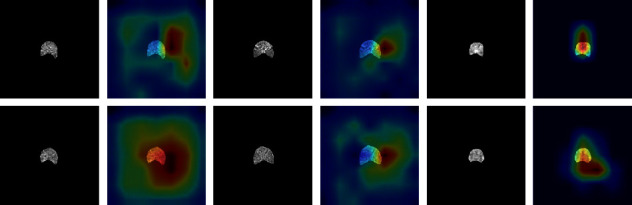
Grad-CAM visualization of 3 cases. From left to right are osteoporosis, osteopenia, and normal cases. The first line represents the L1 vertebral body, and the second line represents the L2 vertebral body.

We further use gradient weighted class activation mapping (Grad-CAM) to visualize the decision information of the feature extraction module.  Figure 11 shows that the feature extraction modules for different categories (normal, osteopenia, and osteoporosis) focus on different regions, and the model automatically focuses on the corresponding regions. Compared with the correctly classified decision information, we also list some cases of misclassification in Figure 12. The focus area of the wrong case has changed significantly compared with the correct case in Figure 11, which may be used as an explanation for the neural network decision error.

Meanwhile, we calculated that the AP value of all testing datasets in the positioning task is 95%, the average IOU in the segmentation task is 0.972 ± 0.125, and the average Dice is 0.983 ± 0.036, which shows that we have good efficiency in selecting features in the positioning and segmentation tasks, but in some cases, these features have no good effect on classification.

#### 4.5.4. Ablation Experiments

In this section, we conduct an ablation study (learning rate is 10*e* − 3) of our method to prove the effective impact of segmentation feature and classification feature layered fusion (LF), gated convolution (GC) module, and feature fusion module (FF).

**Figure 13:**
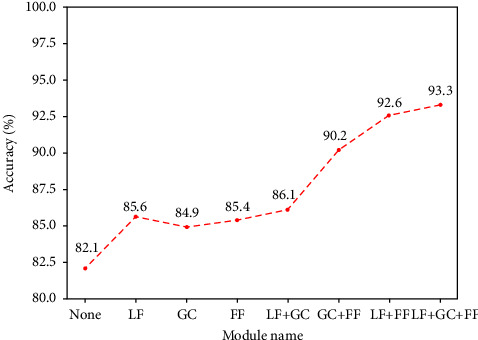
The overall accuracy line chart of the ablation experiments.

We use the three modules separately and combine them randomly and calculate the overall accuracy of each experiment to evaluate whether the model is improved. The quantitative result can be found in Table 3. In Figure 13, it can be clearly seen that the accuracy of the model has been greatly improved. When we calculate without using the method of three modules; it is unfortunate to find that the accuracy of the model is only 82.1%. However, when we perform a hierarchical fusion of segmentation features and classification features, the overall accuracy rate rises to 85.6%, an increase of 3.5%. When we use the gated convolution module, we find that the accuracy rate has increased by 2.8%. When we use feature fusion of vertebral bodies at different levels, the overall accuracy rate has increased by 3.3%. When we select any two of them, we find that the overall accuracy rate has increased by 4%, 8.1%, and 10.5%, respectively. The seven additional experiments prove the feasibility and effectiveness of our proposed modular methods in improving classification accuracy.

## 5. Conclusion

Machine learning can help a great deal in accurately identifying osteoporosis from CT images. In this study, we propose a joint learning framework for bone state detection, where we integrate positioning, segmentation, and classification into an end-to-end multitask joint learning framework. The framework processes from the original input to the final output, increasing the overall fit of the model. The accuracy of classification has been improved by modular task fusion, global feature association, and fusion of different vertebral features. We used a CT image database containing three categories of vertebrae to evaluate this method. A large number of experiments confirm this method improves the overall accuracy from 82.1% to 93.3%, which shows the effectiveness of joint learning in bone state image classification and contributes to solving the problem of clinical diagnosis of osteoporosis.

## Figures and Tables

**Table 1:** Comparison of joint framework performance at different learning rates.

Learning rate		Accuracy	Sensitivity	Specificity	*F*1-score
0.1	Normal	0.876	0.910	0.813	0.862
	Osteopenia	0.816	0.818	0.810	0.865
	Osteoporosis	0.935	0.951	0.823	0.963

0.01	Normal	0.936	0.955	0.923	0.925
	Osteopenia	0.898	0.905	0.880	0.927
	Osteoporosis	0.962	0.983	0.829	0.978

0.001	Normal	**0.971**	**0.964**	**0.976**	**0.964**
	Osteopenia	**0.933**	**0.970**	**0.836**	**0.954**
	Osteoporosis	**0.957**	**0.962**	**0.922**	**0.975**

0.0001	Normal	0.880	0.964	0.825	0.866
	Osteopenia	0.811	0.848	0.716	0.866
	Osteoporosis	0.921	0.921	0.922	0.953

0.00001	Normal	0.900	0.941	0.873	0.882
	Osteopenia	0.864	0.868	0.853	0.902
	Osteoporosis	0.964	0.981	0.843	0.980

The bold value indicates that this is the best model results.

**Table 2:** Comparison with the state-of-the-art baselines on dataset.

Models		Accuracy	Sensitivity	Specificity	*F*1-score
AlexNet (2012) [43]	Normal	0.868	0.785	0.785	0.878
	Osteopenia	0.799	0.793	0.793	0.687
	Osteoporosis	0.931	0.882	0.882	0.756

VGG-19 (2014) [44]	Normal	0.856	0.994	0.765	0.847
	Osteopenia	0.756	0.795	0.655	0.825
	Osteoporosis	0.900	0.894	0.941	0.939

GoogLeNet (2015) [45]	Normal	0.899	0.976	0.849	0.886
	Osteopenia	0.811	0.871	0.655	0.869
	Osteoporosis	0.911	0.902	0.980	0.947

ResNet-50 (2016) [41]	Normal	0.911	0.874	0.936	0.888
	Osteopenia	0.868	0.894	0.802	0.907
	Osteoporosis	0.956	0.995	0.986	0.976

ResNet-101 (2016) [41]	Normal	0.938	0.958	0.924	0.925
	Osteopenia	0.871	0.917	0.750	0.911
	Osteoporosis	0.933	0.940	0.882	0.961

DenseNet-121 (2017) [46]	Normal	0.897	0.982	0.840	0.884
	Osteopenia	0.849	0.841	0.871	0.889
	Osteoporosis	0.952	0.967	0.843	0.972

ShuffleNet (2018) [47]	Normal	0.926	0.970	0.896	0.913
	Osteopenia	0.856	0.911	0.716	0.902
	Osteoporosis	0.931	0.924	0.980	0.959

EfficientNet (2019) [48]	Normal	0.926	0.976	0.892	0.913
	Osteopenia	0.871	0.904	0.784	0.910
	Osteoporosis	0.945	0.943	0.961	0.968

Joint framework (ours)	**Normal**	**0.971**	**0.964**	**0.976**	**0.964**
	**Osteopenia**	**0.933**	**0.970**	**0.836**	**0.954**
	**Osteoporosis**	**0.957**	**0.962**	**0.922**	**0.975**

The bold value indicates that this is the best model results.

**Table 3:** Ablation experiments in a joint learning framework.

LF	GC	FF		Accuracy	Sensitivity	Specificity	*F*1-score
			Normal	0.873	0.743	0.960	0.824
			Osteopenia	0.825	0.921	0.578	0.884
			Osteoporosis	0.943	0.978	0.686	0.967
			Normal	0.907	0.784	0.988	0.870
✓			Osteopenia	0.859	0.957	0.603	0.907
			Osteoporosis	0.947	0.970	0.784	0.970
			Normal	0.889	0.802	0.948	0.853
	✓		Osteopenia	0.849	0.921	0.664	0.898
			Osteoporosis	0.959	0.983	0.784	0.977
			Normal	0.907	0.892	0.916	0.884
		✓	Osteopenia	0.854	0.900	0.733	0.899
			Osteoporosis	0.947	0.965	0.824	0.969
			Normal	0.904	0.850	0.940	0.877
✓	✓		Osteopenia	0.861	0.924	0.698	0.906
			Osteoporosis	0.956	0.972	0.843	0.975
			Normal	0.935	0.982	0.904	0.924
	✓	✓	Osteopenia	0.904	0.901	0.914	0.934
			Osteoporosis	0.964	0.978	0.963	0.979
			Normal	0.966	0.946	0.980	0.958
✓	✓		Osteopenia	0.928	0.980	0.793	0.952
			Osteoporosis	0.956	0.956	0.961	0.972

## Data Availability

The data used to support the study are included within the article.
